# Data-Driven Commissioning to Reduce Type 2 Diabetes Related Health Disparities in The Netherlands: Using Key Informant Group Interviews

**DOI:** 10.3390/healthcare14121621

**Published:** 2026-06-09

**Authors:** Els Roorda, Marc Bruijnzeels, Jeroen Struijs, Marco Spruit

**Affiliations:** 1 Department of Public Health and Primary Care (PHEG), Leiden University Medical Centre (LUMC), 2300 RC Leiden, The Netherlands; m.a.bruijnzeels@lumc.nl (M.B.);; 2 Dutch Healthcare Authority, 3584 BX Utrecht, The Netherlands; 3 Leiden Institute of Advanced Computer Science (LIACS), Leiden University, 2300 RA Leiden, The Netherlands

**Keywords:** health policy, health inequalities, business intelligence, data and analytics

## Abstract

**Highlights:**

**What are the main findings?**

- This explorative study adds the fifteen factors influencing commissioners’ ability to use data to reduce health disparities in type 2 diabetes and shows that commissioners experience barriers in actionable perspectives rather than in data capabilities.
- Additionally, it identifies three themes containing paradoxical mechanisms that hinder data-driven commissioning: equality, where efforts to ensure equal access lead to unfavorable unequal outcomes; quality, where a focus on evidence-based healthcare interventions limits effectiveness in reducing health disparities; and organizational sustainability, where conservative organizational behavior threatens long-term viability.

**What are the implications of the main findings?**

- Current policy often focusses on data capabilities rather than the ability to implement data-driven approaches.
- To limit the rising healthcare usage associated with disparities, policies must be designed to break the paradoxical mechanisms. Further policy analyses are needed to better understand which conditions facilitate commissioners.

**Abstract:**

Background: Health disparities in individuals with type 2 diabetes result in an enormous amount of healthcare usage and are difficult to address. We examine how Dutch private, not-for profit health insurers, responsible for accessibility of care, use their claims data in the commissioning process to reduce health disparities. Objective: To identify factors influencing the possibility to reduce health disparities in commissioning based on data-driven insights. Methods: Key informant group interview data was analyzed using a hybrid deductive–inductive approach following the 6SQIuD framework to identify factors, their relationships, and potential for change, with results validated using a saturation, member and expert check. Results: From the 79 factors found, three were data capability-related and 76 were decision context-related. Fifteen main factors were found on socio-cultural, system, organizational and interpersonal levels. The factors in the decision context can be divided into the themes equality, quality and organizational sustainability. Conclusions: This study explored the factors influencing commissioners’ ability to reduce disparities and the role of data. Interestingly, no main factors related to data capabilities were identified. Three paradoxes were seen after interpreting our data: equal access leads to unfavorable unequal outcomes; a focus on evidence-based healthcare interventions limits effectiveness in reducing health disparities; and conservative organizational behavior threatens long-term viability. Further policy analyses are needed to better understand which systemic and organizational conditions facilitate commissioners in addressing health disparities.

## 1. Background

Health disparities among individuals with type 2 diabetes (T2D) place a significant financial burden on the healthcare system [1]. We calculated that the additional costs associated with health disparities for low-income individuals with T2D is nearly 5% of the total annual healthcare expenditures in The Netherlands. Insurance claims data showed that T2D prevalence was substantially higher among low-income individuals (8.5% vs. 3.2%), who also had higher annual healthcare costs (€9175 vs. €5798) compared to high-income individuals with T2D (see Supplementary Material S1 for more information) [2]. Solving health disparities is complex [3]. While current efforts to solve disparities often focus on individual sectors, such complex challenges demand integration across sectors, including financial alignment [4]. In change literature, such transformation is described as a third-order change or a wicked problem [5,6,7,8]. Different stakeholders often hold fundamentally different views regarding the causes of and solutions to and even the definition of wicked problems. Rittel states that one cannot meaningfully search for information without the orientation of a solution concept [8]. Therefore, in this article, we aim to explore the relationship between health inequalities and data-driven commissioning as a solution.

The international literature shows that healthcare financing and contracting, like copayments or value-based care, can influence health equity [9]. The Dutch healthcare system under investigation operates within a regulated market system, where commissioners, internationally known as purchasers, from insurers and municipalities are responsible for allocating funds, while constrained by policy rules that define the boundaries of their actions. Insurers act upon the Health Care Insurance Act and Long-Term Care Act. The literature recognizes that addressing disparities through fragmented commissioning is complex [9,10,11]. In 2010, bundled payments for diabetes care in the Health Care Insurance Act were introduced to reduce fragmentation. Though not intended to reduce disparities, over a decade later, large outcome gaps persist [12]. Municipalities act upon other acts, responsible for public health and social care and benefits. The Integrated Care Agreement (2022), the Healthy and Active Living Agreement (2023), and the Supplementary Care and Wellbeing Agreement (2025) have been established to enable a more integral approach for the upcoming years [13,14,15]. Various factors influence the ability to reduce health disparities, while causes of disparity often lie in the social and physical environment, education, financial situation, health literacy and cultural factors [16,17].

Multiple authors showed the relevance of data-driven approaches to reduce healthcare usage and disparities by identifying problems and underlying causes and designing effective interventions [18,19,20]. Stakeholders across initiatives in the Netherlands experienced a lack of sufficient data [21]. Interestingly, Dutch health insurers possess extensive claims data that can potentially support efforts to reduce health disparities. Jager et al. found that commissioners highly value data in their decision-making processes [22]. Data-driven insights help them explore policy options, navigate complex challenges, justify their decisions, and persuade stakeholders to support and implement proposed strategies [23].

Despite the increasing availability of claims data, its use in lowering health inequalities remains limited. Our earlier research showed that a successful data-driven approach requires both an understanding of data capabilities (the how) and the decision context (the what for) [24]. The ability to use data depends on various factors at different levels—to provide some examples: cultural (is it normal to use data?), systemic (is it allowed to use data?), organizational (is it possible to get the right data?), and (inter)personal (is there a willingness to use data?).

Reducing health disparities can be considered a wicked problem that may require unequal investments to achieve more equal outcomes. At present, it remains unclear which barriers commissioners experience when attempting to use data-driven approaches to address these disparities. Such insight is needed to determine what further knowledge is required to support approaches such as Population Health Management, in which additional efforts are directed toward groups facing elevated risks. Or as Rittel states: “One cannot understand the problem without knowing about its context” [8]. Therefore, this study explores which factors influence the possibilities for reducing health disparities through data-driven commissioning.

## 2. Methods

Key informant group interviews were used because they enable in-depth exploration of complex phenomena within wicked problems, where problem definitions, scope, causes and potential solutions are often ambiguous and perspective-dependent [8]. Experienced healthcare commissioners were selected as key informants because of their central role in commissioning decisions. To provide structure to the content of the interviews, the 6SQuID framework is used, while the first steps align with the concepts from wicked problem theory stating that problem definition, scope, causes and solutions are related to perspective [25]. The framework consists of six steps, with the first three aimed at systematically mapping factors and identifying opportunities for change:

1. Define and understand the problem and its causes.
2. Clarify which causal or contextual factors are malleable and have the greatest scope for change.
3. Identify how to bring about change: the change mechanism.

Step 4 to 6 are out of scope while describing the design and implementation of an intervention. Steps 1 and 2 were discussed in two separate group interviews with the same design. Next, based on the outcomes of the interviews, the change mechanism (step 3) was discussed in two iterations, first only with experts then in a broader group consisting of the experts and a subset of the interviewees, assuring validity by using a member and expert check [26].

### 2.1. Key Informant Group Interviews

Two 2 h group interviews were conducted via Microsoft Teams, each involving 5–7 participants. The virtual setup facilitated the participation of geographically dispersed individuals and ensured that records of the discussions were maintained for further analysis. Two moderators were involved: the primary moderator asked the prepared questions, summarized parts of the discussion, and asked follow-up questions, while the secondary moderator also asked probing questions and afterwards revised the automatically generated Microsoft Teams transcripts by relistening to the recordings. A semi-structured format was employed based on steps 1 and 2 of the 6SQuID framework (see Supplementary Material S2). This format was developed by the moderators and reviewed by the co-authors.

The discussions began with an exploration of the current process (step 1), aiming to understand how healthcare commissioning for T2D operates presently. Participants were asked about the actions currently taken while using data in commissioning care considering health disparities, and to identify the success factors and barriers they experience in this process.

Subsequently, the focus shifted to envisioning an ideal commissioning process free of existing barriers, with a particular emphasis on addressing health disparities. Participants conceptualized the actions that should take place in the future of commissioning care and discussed the potential role of data and information. The discussions also addressed which factors (step 2) are malleable and how they interact. Finally, participants considered how data can be used to support this ideal process.

After the group interviews we explored how to bring change (step 3 of 6SQuID). At first, the authors discussed practical approaches feasible for implementation, in collaboration with the Ministry as sponsor and subject matter expert. The summary of findings and proposed approaches were discussed in a second meeting with several interviewees.

### 2.2. Participants

For the groups interviews individuals holding the positions of commissioner or policymaker from the seven largest health insurance corporations were invited. To ensure we reached the most relevant participants, we consulted ten experts in the field to gather contact information. These potential participants were approached via email to confirm if they were indeed the appropriate person for participation or to identify a more suitable colleague. All participants were selected based on their affinity with T2D. The invitations included information on the research background, the presence required, the rationale for their selection, and a request to confirm if they or a colleague could attend. After the initial round of invitations, efforts were made to recruit at least one participant from each insurance company through professional networks to ensure comprehensive representation. Attending participants gave written informed consent prior to the interviews.

### 2.3. Data Analysis

A hybrid deductive–inductive content analysis approach was used to code and analyze the current and ideal situations of the decision context and data capabilities [27]. Transcripts were uploaded to the ATLAS.ti software V25 and independently coded by two investigators using the a priori codes that can be found in Supplementary Material S3. Quotes describing the current and ideal situations were labeled like the factors. Factor codes were based on the first steps of the 6SQuID framework. The quotes coded as factors were divided into decision context or data capabilities and were clustered inductively by the authors under main factors; these main factors were proven in the expert sessions, leading to improved main factors. These main factors were clustered under dilemmas. A two-person consensus approach was used to resolve coding discrepancies.

The current and ideal situation and the main factors found were summarized in a presentation for the group of experts and sponsors from the Ministry. The presentation was discussed in a meeting. Adjustments were made to the presentation, and it was discussed again in a second meeting with the same group, supplemented by a number of interviewees.

## 3. Results

### 3.1. Participants

As no data exist on the number of healthcare commissioners involved in T2D care in the Netherlands, we estimated the population size using two extrapolation methods, resulting in an estimated total of approximately 300 (method 1: 270, method 2: 311) commissioners. A total of 35 individuals, approximately 10% of the total population, were invited; from this group thirteen accepted the invitation, seven did not respond, six declined due not seeing themselves as the right candidate and nine declined due to other reasons like a lack of time.

The thirteen participants had the following characteristics: five female and eight male. All participants have worked multiple years in the health insurance business (average 10 years), for one or multiple health insurance firms (average 1.4). The market share of the firms they worked for is 97%. Eight participants have experience in healthcare commissioning and five in policy making for commissioning; one participant also has experience as a data-analyst and one as a medical advisor for commissioners.

The first expert meeting consisted of six participants, three scientists, two policymakers from the Ministry and one data expert. The second expert meeting had ten participants, four commissioners, three scientists, two policymakers from the Ministry and one data expert.

#### 3.1.1. Step 1: Defining and Understanding the Problem and Its Causes

In the current healthcare commissioning landscape only temporary funding is used to finance several projects aiming to lower health disparities. For example the Combined Lifestyle Intervention (in Dutch: GLI) for individuals with a high risk of cardiovascular complications. A participant described that the GLI tends to be more effective for individuals with higher health literacy, so despite its benefits this project potentially increases health disparities. The general consensus is that there are just a few examples of insurers commissioning services with the explicit goal of reducing health disparities. Especially in secondary care, commissioning is rarely focused on disease-specific or population-based approaches due to a lack of time.

*“In essence, our focus in commissioning is not on reducing health disparities. It remains a challenge to identify a viable approach to address disparities.”*

*“What I find particularly complex is that specialist medical care consists of 23 specialties, and the costs associated with diabetes care are actually quite small in comparison. You have to choose how to spend your time.”*

Currently, one insurer compiles data for primary care groups to provide insights into healthcare utilization and inclusion in the diabetes care chain. This data serves as a benchmark based on age, gender, and SES, including expected inclusion rates, enabling comparisons between care groups and regions. It is the responsibility of the care group to determine whether these variations are explainable practice variations without consequences for commissioning. Other insurers are using data to a lesser extent. Furthermore, participants find that it is difficult to determine when an intervention is effective enough and whether this decision is made by the National Healthcare Institute alone or also to some extent by care groups and insurers.

*“You might wonder with several data-supported initiatives: when is it good enough? And who gets to decide, the National Healthcare Institute, or can we decide ourselves?”*

Insurers have both Health Insurance Act and Long-Term Care Act data and are capable of analyzing the health disparities separately; however, they are not allowed to link the data to gain a more integrated view of care. In the context of secondary care, data is used to focus on specific conditions, but when viewed in isolation, the impact is too small to commission effectively. For example T2D care is commissioned in a fragmented manner by multiple departments of insurers (primary care, medical devices and secondary care) and social care is commissioned by municipalities.

#### 3.1.2. Step 2: Clarify Which Causal or Contextual Factors Are Malleable and Have Greatest Scope for Change

Assuming an ideal scenario, participants outlined that payers would be able to allocate their financial resources to the points in the system where the greatest health gains can be realized. This approach ensures that every euro spent contributes maximally to improving health outcomes. The use of data could support this process by identifying which target groups would benefit the most, developing the most fitting intervention strategies for these target groups based on their characteristics, balancing intervention reach and effectiveness and monitoring the impact of interventions after implementation.

*“Ideally we should first collectively determine what we want. What do we want to do differently? Only then should we incorporate the data. What kind of data do we need? And then organize accordingly.”*

#### 3.1.3. Causal and Contextual Factors

**Figure 1:**
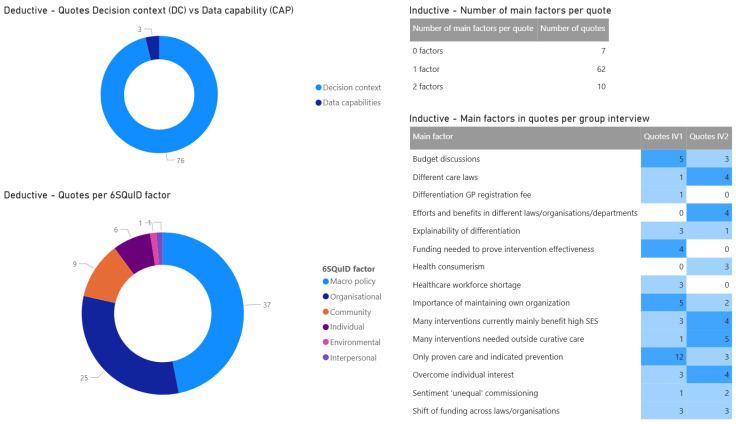
Results of the coding process.

A total of 79 quotes containing factors were identified through deductive coding, as shown in Figure 1, of which 76 related to the decision context and three to data capabilities. Most factors were categorized at the macro policy (37) or organizational (25) level. Of the coded quotes, 62 contained one main factor, 10 contained two main factors, and seven contained no main factor. All main factors found with inductive coding concerned the decision context.

*“It is naive to think that as insurers, we lack the data capabilities to gain insights into health disparities. The issue lies not in obtaining the data, but in the actionable perspectives we can develop thereafter.”*

Ten main factors were discussed in both group interviews, while three main factors emerged only in the first focus group and two only in the second, suggesting reasonable thematic saturation. In both interviews, the final factors emerged after approximately 1.5 h, suggesting that the discussions had largely reached closure. In the first interview, 13 of the 15 factors were identified, while the second interview yielded only two additional factors, suggesting reasonable thematic saturation. No additional factors emerged during the expert session. As complete saturation was not the primary aim given the exploratory nature of the study, the achieved level of saturation was considered sufficient for the study objectives. Supplementary Material S4 contains the factor quotes, the deductive codes and main factors.

#### 3.1.4. Socio-Cultural Factors (Environmental and Community)

The socio-cultural factors found influencing health disparities are healthcare workforce shortage and health consumerism. The concept of consumerism in healthcare implies that individuals expect value for their premiums, claiming their right to care. Workforce shortages result in longer waiting times. A participant argued that high-income groups more often show consumerism, increasing health disparities.

#### 3.1.5. System-Level Factors (Macro Policy)

Factors on the system level are different care laws, a shift in funding across laws and organizations, explainability of differentiation, differentiation of GP registration fees and that only proven care and indicated prevention can be commissioned. Different healthcare laws create silos, limiting a holistic approach to health disparities. A shift in funding is needed between ministry departments, laws and organizations to decrease health disparities.

*“So if the Ministry of Health really has the courage, they won’t organize this from within the ministry alone. Healthcare accounts for only 10% of resilience—the rest lies with the Ministry of Finance, the Ministry of Housing, and so on—it’s a vast societal challenge.”*

Additionally, allocating more resources to certain providers to address health disparities can create tensions with other healthcare providers who receive fewer resources. For example, a legal challenge arose when an insurer adjusted its GP support model based on population characteristics, highlighting the importance of explainability. General practitioners are already compensated for serving low-SES patients through elevated capitation fees, which complicates explainability of further differentiation. Another challenge is that the Health Insurance Act supports only proven care and indicated prevention, limiting opportunities for innovative preventive measures.

*“We cannot invest in general prevention as an insurer, but we are very interested in targeted prevention for specific groups.”*

*“We have undertaken projects where we have observed that forgiving debts or even purchasing debts can help reduce health costs. Technically, we are not allowed to do that. There is a distinction between the formal role and the informal role or what is sensible. In fact, as a health insurer, we are supposed to commission healthcare.”*

An interviewee emphasized the need for a common understanding and that coordinated effort is needed to address systemic issues.

*“We need a shared goal, but more importantly, we must identify where the problems lie and work together to solve them.”*

#### 3.1.6. Organizational Factors

Organizational factors found are the importance of maintaining one’s own organization, efforts and benefits in different laws/organizations/departments, many interventions mainly benefitting high SES, funding being needed to prove intervention effectiveness and many interventions needing outside curative care. At the organizational level, the influence of systemic factors became visible.

Within organizations, maintaining the status quo often hinders innovation and efforts to reduce health disparities. Preserving existing structures can block new practices, as seen in ministries, insurers, healthcare providers and medical specialists. For instance, despite longstanding efforts in diabetes care, the number of internists has not decreased. A pilot showed that a glucose sensor was more effective than a dietician but was not adopted due to financial penalties for general practitioners. In contrast, a cardiology pilot demonstrated that changing the financial model increased focus on preventive care by reducing the financial risk to organizations, highlighting the need to coordinate the transition.

*“I think that if we really want to make progress in reducing health disparities, there needs to be a clear problem owner who takes the lead in identifying key influence points and opportunities, and who actively engages and encourages the various stakeholders to act on them.”*

Most interventions tend to be more effective for individuals with high SES. However, the system prescribes that only proven care and indicated prevention can be funded, posing a significant barrier to fund innovative approaches tailored to low-SES populations.

*“When I was still working as a researcher, many colleagues were very frustrated because their goal was to reduce health disparities. However, every project they undertook seemed to only increase these differences, as they consistently reached only highly educated individuals, no matter how effective, how appealing, how well-designed the interventions were. So, that is indeed a significant challenge.”*

Another factor is that funding is needed to develop interventions that reduce health disparities. Developing and scientifically validating new interventions requires substantial investment, creating a classic “chicken and egg” problem: proving the effectiveness of these interventions is necessary for funding, but funding is needed to establish their effectiveness.

*“The challenge lies in the fact that funds can only be allocated to proven interventions, yet implementing an intervention is necessary to gather the evidence of its effectiveness.”*

#### 3.1.7. Interpersonal (Individual and Interpersonal)

Interpersonal factors are: overcoming individual interest, budget discussions and the sentiment of ‘unequal’ commissioning. Designing and making agreements is a process carried out by individuals in consultation with one another. Significant adjustments to budgeting can lead to extensive discussions and disagreements, impacting the individuals responsible for negotiating agreements. These adjustments can create tensions among stakeholders, including healthcare providers and insurers, as they navigate the complexities of funding redistribution and its implications for service delivery.

Another factor is the sentiment among premium payers who may feel that they are being treated unfairly if others are perceived to have access to specific care that they do not. This sense of inequity can arise when interventions are tailored to specific populations, potentially leading to dissatisfaction and resistance from those who feel excluded.

#### 3.1.8. Relations Between Main Factors

**Figure 2:**
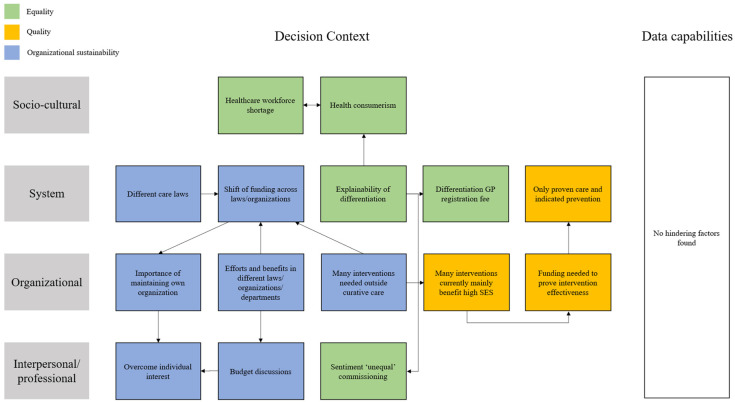
Overview of the main factors and their interrelationships hindering a data-driven commissioning to reduce health disparities discussed in the group interviews, categorized by type (blue, green, yellow) and level (in grey).

The main factors and their relations are summarized in Figure 2. Three related clusters were found. The first cluster in green relates to equality. Healthcare workforce shortages create scarcity, requiring patients to navigate the system effectively to receive timely care. Introducing different approaches requires explaining differentiation, which is complex due to existing compensation for GPs treating low-SES patients. This may lead to professionals feeling short-changed. The second cluster in yellow relates to quality. The current system focuses on proven care and indicated prevention, which tends to be more effective for higher SES groups. Developing alternative interventions is challenging due to funding barriers. The third cluster in blue concerns organizational sustainability. Reducing health disparities requires different care models and shifting financial resources, even when costs and benefits arise in different sectors. Many organizations prioritize self-preservation, leading to discussions and the need to overcome individual interests.

#### 3.1.9. Step 3: Identify How to Bring About Change: The Change Mechanism

In the first expert session, we concluded that socio-cultural factors are difficult to change, while systemic changes take time. However, the Integrated Care Agreement creates opportunities for temporary cross-sector interventions, using data to justify unequal investment and quantify necessary efforts and outcomes. The Integrated Care Act provides some scope for testing new approaches beyond healthcare, but ensuring sustainable financing remains a challenge.

Healthcare and welfare organizations, united in regional collaborations, are best positioned to implement interventions, while insurers have the most comprehensive data to identify problems and potential impacts. This reveals a gap between data capabilities and the decision-making context. Next, in the expert sessions, we discussed who should take action to bridge this gap. The outcome was that commissioners can share data with regional collaboration platforms, enabling partner organizations to act upon it. For policy purposes, an animation was then created to inspire commissioners to adopt this approach more frequently [28].

## 4. Discussion

Health disparities in type 2 diabetes account for a substantial share of healthcare usage and expenditures in the Netherlands. Commissioners are responsible for allocating health funds and ensuring accessibility. Solving disparities with commissioning based on data is a multidimensional wicked problem, a completely new research field and there is no definitive problem formulation [8]. To get a better understanding of the problem, we explored factors from a commissioning perspective using the first steps of the 6SQuID method, showing its value in understanding stakeholder perspective on big systems beyond its traditional application in intervention development [25]. Surprisingly, we identified 76 factors within the decision context, of which 37 were related to the policy level, while only three concerned data capabilities. This contrasts with Population Health Management initiatives in The Netherlands focusing on health equity, which frequently report barriers related to data capabilities [21]. This may be explained by the different stakeholder perspective examined in this study, namely commissioners rather than data analysts, as commissioners’ capabilities, role and working environment were found as a factor influencing data usage by Jager et al. [22]. On the side of the decision context, three groups of factors were identified, linked to equality, quality, and organizational sustainability. We concluded that the informants’ perspectives and understanding of unequal care and outcomes reflect broadly shared but limited or even false perception of paradoxes:

- Equality paradox: Efforts to ensure equal access lead to unfavorable unequal outcomes.
- Quality paradox: A focus on evidence-based healthcare interventions limits effectiveness in reducing health disparities.
- Organizational sustainability paradox: Current conservative organization behavior threatens the long-term viability of organizations.

Examining the underlying mechanisms, the equality paradox arises as labor shortages increase pressure on healthcare services, necessitating greater health literacy for timely access. Lower SES groups face greater challenges in finding their way through the system while they often need more support to achieve similar outcomes [10,29]. Whitehead stated, regarding equity and access: “Equal access to available care for equal need, equal utilization for equal need, equal quality of care for all.” [30]. In our data, however, we observed both a higher prevalence of T2D and more complications among lower-income groups. From a purely medical perspective, high- and low-income individuals with T2D may be considered to have equal needs, yet they experience unequal outcomes under the same guidelines and care structures. This raises the question whether Whitehead’s concept of “equal need” should be interpreted more broadly, incorporating social determinants of health into medical guidelines and care pathways as suggested by Kist et al. [20]. Looking at the Dutch health agreements, the equity paradox is absent in the first agreements in 2022/2023, but is partly acknowledged in the new agreement in 2025 which states: “More equitable access to healthcare, particularly between higher-educated individuals who are better able to navigate the system and those for whom this is more difficult.” [13,14,15].

In the quality paradox, healthcare funding is restricted to proven interventions, which currently primarily benefit higher SES groups, while it remains unclear which interventions are effective for lower SES populations. However, they likely require a more multidisciplinary approach, where healthcare and social services are combined, rather than traditional healthcare interventions [16,31]. Limited investment in yet unproven interventions slows the development and implementation of new effective solutions for low-SES groups, unintentionally reinforcing inequality. Current finance models lack incentives to shift towards preventive strategies; providers and payers even face financial risks when shifting towards them, leading commissioners to act primarily based on financial incentives rather than data-driven insights. This may partly result from the dynamic in which medical guidelines determine reimbursement, while reimbursement structures simultaneously reinforce adherence to existing medically oriented guidelines. As a result, broader social determinants of health may remain insufficiently addressed within care pathways. A pilot project demonstrated that a stronger focus on prevention emerges when healthcare is financed through population-based payments, consolidating accountability for both outcome and expenditures [32,33,34]. This approach is also observed internationally, such as in the Accountable Care Organizations in the United States [35]. Looking at the older health agreements, the Integrated Care Agreement (IZA) mainly focuses on healthcare delivery and the Healthy and Active Living Agreement (GALA) on primary prevention. The new agreement (AZWA) seems to create room for experimenting with and evaluating still-developing interventions through a learning-based approach [13,14,15].

The organizational sustainability paradox highlights that reducing health disparities requires financial reallocation. However, organizations and departments resist losing resources, leading to budget disputes and stalled reforms. As disparities grow, so does financial pressure, ultimately reaching a point where efficiency measures are exhausted, staffing shortages persist and organizations face collapse. Within the IZA and GALA, financial structures largely remain confined to existing sectoral boundaries, whereas the AZWA describes opportunities for cross-domain financing, providing a foundation for further development in this area [13,14,15].

To provide commissioners with actionable information, data capabilities and the decision context must be brought together to support a data-driven approach [24]. The paradoxes found indicate that the main constraint for a data-driven approach lies within the decision-making context, preventing commissioners from effectively reducing the high healthcare utilization associated with T2D-related health disparities. However, in the literature and practice the focus often lies on data capabilities rather than the integration in the decision context [36]. For data-driven approaches to address wicked problems effectively, they therefore need to become part of health policy and organizational strategy, consistent with concepts such as business–IT alignment [37]. Further policy analyses are needed to better understand which systemic and organizational conditions, given these paradoxical mechanisms, facilitate commissioners in addressing health disparities.

## 5. Strengths and Limitations

Data was collected and analyzed in a structured manner using the 6SQuID framework, providing a clear and systematic approach. However, this study has several limitations. First, the group interviews were limited in size and scope, which may not fully capture the diversity of experiences and perspectives among all healthcare commissioners in this new field. The network-based recruitment strategy may also have contributed to this limitation. Nevertheless, during the second interview, only two of the 15 identified themes were newly observed, suggesting a reasonable degree of thematic saturation. Second, the study relied on qualitative data, which, while rich in detail, may be subject to selection, discussion guide and interpretation biases. For example, we deliberately recruited more experienced healthcare commissioners given the complexity of the topic, which may have limited the visibility of potential generational differences in perspectives. Differences in perspectives and roles between commissioners and policymakers involved in commissioning were not explicitly analyzed. Third, the complexity and “wickedness” of the identified problems after step 2 exceeded the scope originally envisioned for the expert sessions. As a result, the planned expert-based reflection became less suitable, while a more extensive policy analysis for each identified paradox would likely have been more appropriate. Consequently, the findings may be less generalizable beyond the studied context. In the current study, we nevertheless effectively conducted a thorough exploratory analysis of the Dutch healthcare agreements to contextualize the discussion.

## 6. Conclusions

This exploratory study was the first to investigate the factors influencing commissioners’ ability to reduce health disparities and the role of data, categorized by decision context and data capability at different levels (socio-cultural, system, organizational, and interpersonal). Interestingly, only three factors related to data capabilities were identified by participants and 76 factors related to the decision context. Three paradoxes were seen after interpreting our data: equality, where efforts to ensure equal access lead to unfavorable unequal outcomes; quality, where a focus on evidence-based healthcare interventions limits effectiveness in reducing health disparities; and organizational sustainability, where conservative organizational behavior threatens long-term viability. These paradoxes hinder commissioners in applying a data-driven approach to reduce health inequalities in type 2 diabetes. Further policy analysis is needed.

## Data Availability

All data generated is available in the publication and Supplementary Materials.

## Supplementary Materials

The following supporting information can be downloaded at: https://www.mdpi.com/article/10.3390/healthcare14121621/s1, Supplementary Material S1: Income related health disparities in Type 2 Diabetes; Supplementary Material S2: Group interview topics; Supplementary Material S3: A priori codes; Supplementary Material S4: Coded factor quotes.
