# Supplementary material for: Data-Driven Commissioning to Reduce Type 2 Diabetes Related Health Disparities in The Netherlands: Using Key Informant Group Interviews
Source: Healthcare (Basel). 2026 Jun 9;14(12):1621. doi: 10.3390/healthcare14121621 (PMC13299924; doi:10.3390/healthcare14121621)
Supplement: Supplementary file 1 [file healthcare-14-01621-s001.zip › Supplementary Material S1.pdf]

Supplementary Material 1: Income related health disparities in Type 2 Diabetes

An analysis examining insurance claims data revealed that the prevalence of T2D is significantly higher among lower-income households, with 8.5% of individuals in the lowest 30% income group, compared to just 3.2% in the highest 30%. On the other hand, among those diagnosed with T2D, average healthcare costs per insured individual amount to €9,175 in the lowest income group versus €5,798 in the highest income group, while insured individuals without T2D in the highest income group have an average cost of just €1,865.

Income group	Population size	Prevalence of DM2	Average claims costs individuals with DM2	Average claims costs individuals without DM2
Lowest 30%	4,751,393	405,899 (8,5%)	€ 9,175	€ 3,185
Highest 30%	6,748,054	217,072 (3,2%)	€ 5,798	€ 1,865
Other	6,628,366	357,552 (5,4%)	€ 7,386	€ 2,371
**Total**	**18,127,813**	**980,523 (5,4%)**	**€ 7,775**	**€ 2,384**

*Table 1: The prevalence of T2D is significantly higher among lower-income households, with 8,5% of individuals in the lowest 30% income group, compared to just 3.2% in the highest 30%. On the other hand, among those diagnosed with T2D, average healthcare costs per insured individual amount to €9,175 in the lowest income group versus €5,798 in the highest income group, while insured individuals without T2D in the highest income group have an average cost of just €1,865. A rough estimation of the costs of health disparities: Higher prevalence + higher costs: 4,751,393 *((8,5%-3,2%)* (€ 9,175-1,865)+ 3,2%*(€ 9,175-€ 5,798)) = € 2,351,768,485. Which is 4,85% of total claims value of € 48,511,275,391.*
