# Supplementary material for: Data-Driven Commissioning to Reduce Type 2 Diabetes Related Health Disparities in The Netherlands: Using Key Informant Group Interviews
Source: Healthcare (Basel). 2026 Jun 9;14(12):1621. doi: 10.3390/healthcare14121621 (PMC13299924; doi:10.3390/healthcare14121621)
Supplement: Supplementary file 1 [file healthcare-14-01621-s001.zip › Supplementary Material S2.pdf]

Supplementary Material 2: Group interview topics

1) Current situation: Basic process → (How does the commissioning process for diabetes care currently work?)
   a) What actions are currently taken to commission care while considering health disparities?
   b) What success factors and barriers are experienced in this process?
2) Current use of data?
3) Desired situation: What is your ideal process (without barriers)? → health disparities
   a) What actions would take place in the future to commissioning care while considering health disparities?
   b) What role can information provision/data play in targeted healthcare commissioning?
      i) Which factors are mallable?
      ii) At what level do they operate (organization/policy and regulation)?
      iii) How can the data be made most accessible and usable?
